# Vitamin D inadequacy in Belgian postmenopausal osteoporotic women

**DOI:** 10.1186/1471-2458-7-64

**Published:** 2007-04-26

**Authors:** Audrey Neuprez, Olivier Bruyère, Julien Collette, Jean-Yves Reginster

**Affiliations:** 1 Department of Epidemiology, Public Health and Health Economics, University of Liege, 4020 Liege, Belgium

## Abstract

**Background:**

Inadequate serum vitamin D [25(OH)D] concentrations are associated with secondary hyperparathyroidism, increased bone turnover and bone loss, which increase fracture risk. The objective of this study is to assess the prevalence of inadequate serum 25(OH)D concentrations in postmenopausal Belgian women. Opinions with regard to the definition of vitamin D deficiency and adequate vitamin D status vary widely and there are no clear international agreements on what constitute adequate concentrations of vitamin D.

**Methods:**

Assessment of 25-hydroxyvitamin D [25(OH)D] and parathyroid hormone was performed in 1195 Belgian postmenopausal women aged over 50 years. Main analysis has been performed in the whole study population and according to the previous use of vitamin D and calcium supplements. Four cut-offs of 25(OH)D inadequacy were fixed : < 80 nmol/L, <75 nmol/L, < 50 nmol/L and < 30 nmol/L.

**Results:**

Mean (SD) age of the patients was 76.9 (7.5) years, body mass index was 25.7 (4.5) kg/m^2^. Concentrations of 25(OH)D were 52.5 (21.4) nmol/L. In the whole study population, the prevalence of 25(OH)D inadequacy was 91.3 %, 87.5 %, 43.1 % and 15.9% when considering cut-offs of 80, 75, 50 and 30 nmol/L, respectively. Women who used vitamin D supplements, alone or combined with calcium supplements, had higher concentrations of 25(OH)D than non-users. Significant inverse correlations were found between age/serum PTH and serum 25(OH)D (r = -0.23/r = -0.31) and also between age/serum PTH and femoral neck BMD (r = -0.29/r = -0.15). There is a significant positive relation between age and PTH (r = 0.16), serum 25(OH)D and femoral neck BMD (r = 0.07). (*P *< 0.05)

Vitamin D concentrations varied with the season of sampling but did not reach statistical significance (*P *= 0.09).

**Conclusion:**

This study points out a high prevalence of vitamin D inadequacy in Belgian postmenopausal osteoporotic women, even among subjects receiving vitamin D supplements.

## Background

Osteoporosis is a chronic, progressive disease characterized by reduced bone mass and microarchitectural deterioration of bone, involving an extensive fragility and a subsequent increase in fracture risk [1].

Vitamin D and calcium are essential components of osteoporosis management. Once vitamin D is absorbed from the diet or synthetised in the skin by the action of sunlight, it is metabolized first in liver to 25-hydroxyvitamin D [25(OH)D] and then in kidney to 1,25-dihydroxyvitamin D [1,25(OH)2D], before becoming biologically active. Then, 1,25(OH)2D interacts with its nuclear receptor (VDR) in target tissues where appropriate biological responses are mediated, in particular to maintain calcium homeostasis by increasing efficiency of intestinal calcium absorption [2]. As a consequence, decreasing 25(OH)D serum concentrations leads to reduced calcium absorptive performance yielding to an increase in PTH concentrations [3]. This leads to an increased bone resorption and accelerated bone loss, by increasing the number and activity of osteoclasts that release calcium from bone. Decreasing bone mineral density (BMD) and bone strength with increased susceptibility to fragility-fracture risk are ultimate consequences of vitamin D deprivation [4-6]. Vitamin D inadequacy has also been implicated as a contributing factor to muscle weakness and propensity to fall, both in active and inactive ambulatory elderly subjects [7,8]. A positive relationship has also been shown between cognitive functioning and vitamin D concentrations, which may also influence the risk of fall and fracture [9].

Sunlight and diet are the two sources of vitamin D. UV-B irradiation is the primary source of vitamin D. Approximately 90% of serum vitamin D is produced endogenously from 7-dehydrocholesterol in the epidermis of the skin, after adequate exposure. However, the capacity of the skin to produce vitamin D declines with aging [4,10,11]. Moreover, environmental factors influence the cutaneous production of vitamin D. Latitude, season, and time of day as well as ozone pollution in the atmosphere diversify the number of solar ultraviolet B photons reaching the earth's surface, and thereby, alter the cutaneous production of vitamin D3 [12].

As a consequence, in the light of the implication of vitamin D deficiency in bone metabolism, osteoporosis prevention guidelines developed by scientific authorities contain recommendations for vitamin D intake and evidence-based treatment of osteoporosis requires vitamin D supplements to maintain adequate plasma concentration [13-15].

The objective of this study is to assess the prevalence of inadequate serum vitamin D concentrations in postmenopausal Belgian women.

## Methods

Belgian postmenopausal women aged over 50 years were included in the study (n = 1195), of which overall 52.5 % had a prior fragility fracture. These women were part of a run-in study aimed at normalizing the calcium and vitamin D status of patients prior to be included in a trial, investigating the anti-fracture efficacy of a new anti-osteoporotic drug. Women were eligible for this study if they had been postmenopausal for at least five years, with at least one fracture confirmed by spinal radiography or a lumbar-spine bone mineral density (BMD) ≤ 0.840 g/cm^2 ^or a femoral neck BMD ≤ 0.600 g/cm^2^. Among the women included in this study, 26.4% already received vitamin D supplements (vitamin D3) and 45.4% got calcium supplementation.

After receiving information from the investigator, and being able to ask questions regarding all aspects of the study, all participants provided written informed consent before enrolment; the study was approved by the Institutional Review Board of the University of Liège, BELGIUM.

Fasting blood and urine samples were collected at baseline, stored at -80°C and centrally analyzed. Assessment of 25-hydroxyvitamin D [25(OH)D] was performed with a commercial radioimmunoassay [DiaSorin (formerly Incstar Corporation), Stillwater, Minnesota, USA]. It consists of a two-step procedure. In a first step, the 25(OH)D and other hydroxylated metabolites are rapidly extracted from serum or plasma with acetonitrile. The extract is then assayed by RIA using a polyclonal antibody with specificity to 25(OH)D. The sample, the antibody and the tracer are incubated for 90 minutes at room temperature (20–25°C). The complexes «antigen-antibody» are separated after 20 minutes incubation at RT with a second antibody precipitating complex. This radioimmunoassay is a competitive binding assay with a limit of detection of 4 ng/ml and a within and between assay precision lower than 8%.

In this particular study, four cut-offs of 25(OH)D inadequacy were fixed : < 80 nmol/L (mild deficiency), <75 nmol/L (moderate deficiency), < 50 nmol/L (severe deficiency) and < 30 nmol/L (very severe deficiency). The laboratory performing the 25(OH)D assessment take part in a 25(OH)D quality scheme such as the International Quality Assessment Scheme for Vitamin D metabolites (DEQAS).

The assay of the intact PTH (hPTH 1–84) was performed with the "N-tact(R) PTH SP IRMA Kit" from DiaSorin. Bone mineral density at the lumbar spine and proximal femur was measured by dual-energy x-ray absorptiometry. The BMD of the two proximal femurs were assessed and the lowest femoral neck BMD was reported. A spine phantom was scanned each morning as a quality control and for instrument calibration.

### Statistical analysis

The primary analysis has been performed in the whole study population and in three different categories of age (50–69 years, 70–79 years and 80 years and more). We also performed analysis according to the previous use of vitamin D supplements and we stratified our analysis according to the type of supplementation: patients without supplementation (group 1, n = 637), patients receiving calcium alone (group 2, n = 241), patients with vitamin D but without calcium supplements (group 3, n = 14), patients with calcium and vitamin D supplements (group 4, n = 302).

Analyses have been stratified according to sunlight exposure. In Belgium (50 degrees latitude North), spring is defined as March through June, summer as June through September, fall as September through December and winter as December though March.

Normality of variables has been confirmed by Kolmogorov-Smirnov tests. Student T tests were used to compare mean values of serum 25(OH)D in women with and without vitamin D supplement. ANOVA was performed to compare mean values of serum 25(OH)D and PTH according to the four groups of supplementation, age groups and seasons. Pairwise comparisons with adjustment for multiple comparisons (Bonferroni) were also made if the ANOVA was significant. Correlation coefficients were calculated to assess the relationship between the variables. All the data were analyzed using STATISTICA (version7.1; StatSoft Inc). P-values < 0.05 were regarded as significant.

## Results

The 1195 women included in this study were aged (mean +/- SD) 76.9 ± 7.5 years. Their mean [SD] serum concentrations of 25(OH)D, calcium, PTH were 52.5 (21.4) nmol/L, 2,37 (0.12) mmol/L and 31.2 (14.9) pg/mL, respectively. The characteristics of the study population are presented in Table 1.

**Table 1:** Baseline characteristics of the 1195 Belgian postmenopausal women.

	Mean ± SD	Range
Age (years)	76.9 ± 7.5	50 – 100
Body mass index (kg/m^2^)	25.7 ± 4.5	13.6 – 44.3
Lumbar bone mineral density (g/cm^2^)	0.767 ± 0.13	0.387 – 1.357
Femoral neck bone mineral density (g/cm^2^)	0.522 ± 0.08	0.211 – 0.953
Serum 25-hydroxyvitamin D (nmol/L)	52,5 ± 21.4	12.5 – 175
Serum Calcium (mmol/L)	2,37 ± 0.12	1.93 – 2.85
Serum intact parathyroid hormone (pg/mL)	31,2 ± 14.9	6 – 174.6

In the whole study population, the lowest serum 25(OH)D concentrations (<30 nmol/L) were observed in 15.9 % of all women. When considering the 50 and 80 nmol/L cut-offs, the prevalence reaches 43.1% and 91.3%, respectively.

The mean [SD] serum 25(OH)D concentrations reached 63 (20) nmol/L in women with vitamin D supplements compared to 48.7 (20.7) nmol/L in women without vitamin D supplements (*P *< 0.001).

When stratified according to calcium and vitamin D supplementation, women who used vitamin D supplements, only or combined with calcium supplements, had higher concentrations of 25(OH)D than non-users : 59.3 nmol/L (group 3) and 63.1 nmol/L (group 4) vs 45.9 nmol/L (group 1) and 56.4 nmol/L (group 2) (*P *< 0.001). The groups 1, 2 and 4 differ statistically with each other (Bonferroni correction – *P *< 0.001).

**Figure 1:**
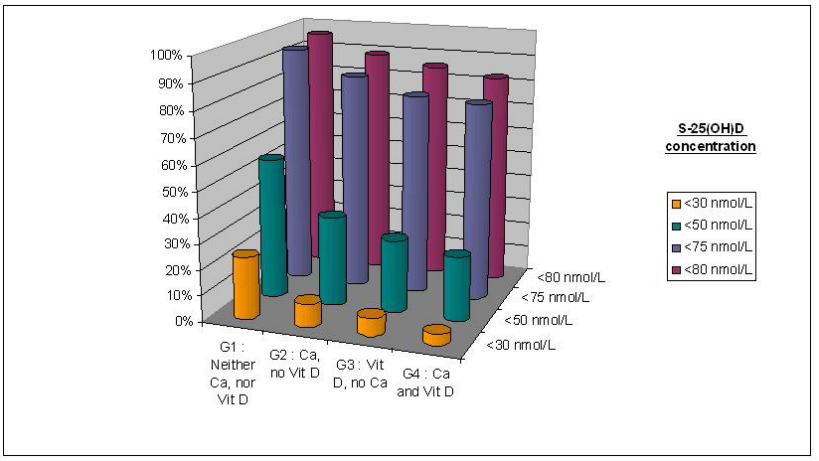
Percentage of subjects having serum 25(OH)D concentration below various thresholds in the different groups stratified according to their type of supplementation.

In groups receiving vitamin D supplements, severe 25(OH)D inadequacy (<50 nmol/L) was reported in 28.6% of the women. Mild and moderate inadequacy was reported by 85.7% and 82.8% of the women, respectively. In absence of vitamin D supplementations, 55.3 % of the patients presented severe inadequacy and 96.2% mild inadequacy (Figure 1).

**Figure 2:**
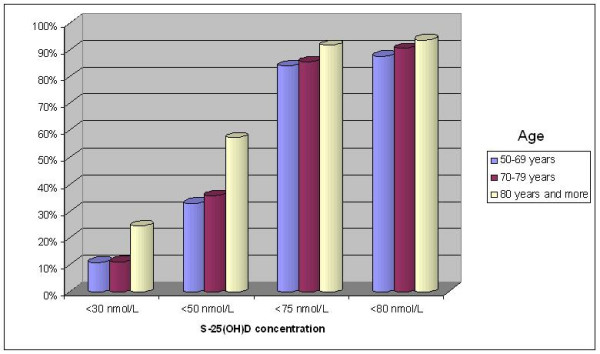
Effect of age on prevalence of vitamin D inadequacy.

There was a significant negative correlation between age and serum 25(OH)D (r = -0.23; *P *< 0.05). The mean serum 25(OH)D concentrations were 57.9 nmol/L in women aged less than 70 years old (n = 137). When considering patients aged more than 80 years (n = 430), 25(OH)D concentrations were 45.7 nmol/L. In the intermediate group (n = 628), the mean concentrations of 25(OH)D were 56 nmol/L. The vitamin D inadequacy was significantly higher in the oldest age group (57.2 % and 93.7 % when considering cut-offs of 50 and 80 nmol/L, respectively) compared with the youngest age groups (32.8 % and 57.6 %) (*P *< 0.001). The youngest age groups differed statistically from the oldest age group (Bonferroni correction – *P *< 0.001). (Figure 2)

**Figure 3:**
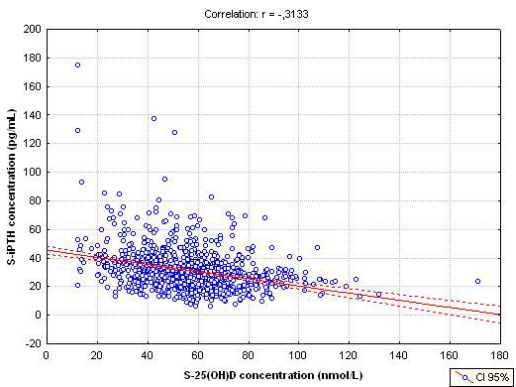
Plot of serum intact PTH concentrations versus serum 25(OH)D concentrations.

**Figure 4:**
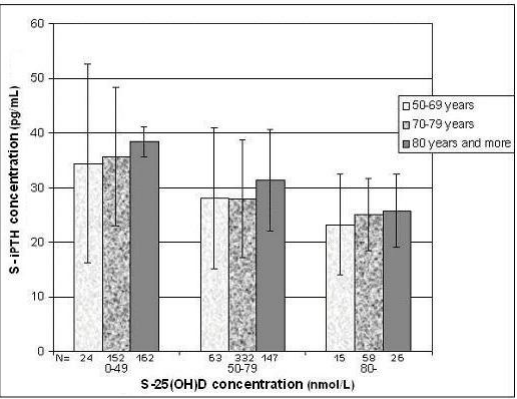
Adjusted mean serum parathyroid hormone values according to serum 25-hydroxyvitamin D values and age group.

A significant negative correlation was also found between serum 25(OH)D and PTH (r = -0.31; *P *< 0.05; figure 3), serum PTH rising with age (r = 0.16;*P *< 0.05). In figure 4, comparing the effects of 25(OH)D concentrations and age on PTH, patients were grouped according to their age category and then further grouped, according to their 25(OH)D concentrations. For all age categories, there was a substantial and progressive decrease of PTH concentration as 25(OH)D increased. The lowest serum PTH concentrations were observed in the groups with serum 25-hydroxyvitamin D concentrations of more than 80 nmol/L, whereas the highest serum PTH was observed in the groups with serum 25(OH)D concentrations lower than 50 nmol/L. The PTH concentrations of the oldest group (80 years and more) were consistently higher than those of younger subjects. These differences are based on significant ANOVA and Bonferroni tests (*P *< 0.001). Furthermore, mean PTH concentrations were statistically different in each category of 25(OH)D concentrations (Bonferroni test – *P *< 0.05)

When stratified according to calcium and vitamin D supplementation, women who used vitamin D supplements, only or combined with calcium supplements, had lower PTH concentrations than non-users: 31.2 pg/mL (group 3) and 26.6 pg/mL (group 4) vs 34.2 pg/mL (group 1) and 30.2 nmol/L (group 2) (*P *< 0.001); although not reaching statistical significance after Bonferroni correction, between group 3 and the three other groups (*P *< 0.05).

A significant negative correlation between age, serum PTH and femoral neck BMD was found in the studied population (r = -0.29 and -0.15; *P *< 0.05). We found also too a positive correlation between serum 25(OH)D and femoral neck BMD (r = 0.07; *P *< 0.05). Whereas no correlation between these variables (age, serum 25(OH)D and serum PTH) and BMD at lumbar spine was observed.

25(OH)D concentration did not vary with the season of sampling. Spring (March 20 – June 21) was characterized by lower concentrations of 25(OH)D (50.9 nmol/L), while higher concentrations were observed in summer (June 21-September 23) (55.1 nmol/L). The prevalence of vitamin D severe inadequacy (50 nmol/L cut-off) during spring, summer, autumn and winter was 45.3%, 39.1%, 44.5 % and 43 %, respectively. However, these values did not reach statistical significance (*P *= 0.09). (Table 2)

**Table 2:** Prevalence of vitamin D inadequacy stratified for seasons.

	1. Spring	2. Summer	3. Fall	4. Winter
<30 nmol/L	15,3%	9,9%	17,5%	20,9%
<50 nmol/L	45,3%	39,1%	44,5%	43,0%
<75 nmol/L	90,8%	86,9%	85,3%	85,4%
<80 nmol/L	93,6%	89,4%	91,0%	90,2%

## Discussion

The results of this study show a high prevalence of vitamin D inadequacy amongst Belgian postmenopausal women, irrespective of the threshold used for the diagnosis.

The serum concentrations of 25(OH)D are most valuable for determining the overall vitamin D status of an individual, since it is an average of dietary and sunlight-induced vitamin D. The measurement of the serum 1,25(OH)2D concentrations has been most useful in evaluating disorders in calcium and bone metabolism related to acquired and inborn errors in the conversion of 25(OH)D to 1,25(OH)2D [16-18].

Assessment of 25-hydroxyvitamin D [25(OH)D] was performed with a commercial radioimmunoassay (DiaSorin). It is important to specify that interlaboratory variability in serum 25-hydroxyvitamin D (25(OH)D) results is often reported because of the use of different methodologies (protein binding, chemiluminescent and RIA assays). This variation in measurement confounds the diagnosis of vitamin D insufficiency. Consequently, these assays must be internationally standardized and made available to practicing clinicians [19]. From this perspective, the International Quality Assessment Scheme for Vitamin D metabolites (DEQAS) was introduced in 1989 to improve the reliability of 25-hydroxyvitamin D (25-OHD) assays [20].

No universal consensus has been reached regarding serum values constitutive of inadequacy. Indeed, the vitamin D status that fully normalizes calcium absorption is not known for humans [3]. Recent publications suggest that the circulating concentration of 25(OH)D should be over 80 nmol/L or at least between 50 nmol/L and 80 nmol/L to optimize intestinal calcium absorption, minimize increased parathyroid activity and reduce the risk of hip and other non vertebral fractures [21,22]. It has been demonstrated that calcium absorption is reduced in patients with 25(OH)D concentrations below 80 nmol/L corresponding to higher PTH secretion (reported at 25(OH)D values below 80 nmol/L) [3]. In this particular study, two cut-offs of 25(OH)D inadequacy were fixed : < 80 nmol/L and < 50 nmol/L. However, we also used the threshold of 75 nmol/l, suggested by a consensus expert panel and a review [22,23] and a concentration of 30 nmol/L, considered by certain authors as the lower limit indicating a severe deficiency [24-27].

Various studies found that postmenopausal women who have osteoporosis (involving low bone mineral density and/or history of fragility fracture) are more likely to have serum vitamin D concentrations below the normal range. For example, in United Kingdom, in 330 patients with fragility fractures from Glasgow, Belfast and Medway, mean concentrations of 25(OH)D ranged from 40 nmol/L to 52.3 nmol/L and 83.7 to 96.4 % of patients had 25-hydroxyvitamin D concentrations < 80 nmol/L and 55.8 to 73.2 % < 50 nmol/L. 694 patients with hip fractures were specially identified from Belfast, Glasgow and London. Their mean concentrations of 25(OH)D ranged from 24.7 nmol/L to 36.1 nmol/L and 81.6 to 92.7 % had a concentration < 50 nmol/L [28]. In a systematic review of 30 publications reporting prevalence estimates for vitamin D inadequacy in populations with osteoporosis associated with other disorders possibly, the prevalence of 25(OH)D concentrations < 12 ng/mL (30 nmol/L) ranged from 12.5 % to 76 %, while prevalence rates reached 50 % to 70 % of patients with a history of fracture(s) using a cut-off of 15 ng/mL [29].

However, the prevalence observed in our cohort of osteoporotic postmenopausal women is generally lower than previously reported in elderly subjects in previous studies and in healthy adults [30]. The women enrolled in our study were fairly healthy, free of diseases other than osteoporosis. In addition, volunteers for clinical trials may be more conscious about their health than the general population. All these factors may have contributed to the lower overall prevalence of vitamin D deficiency observed in this study compared with previous studies. Same observations have been reported in two recent studies. In a multinational study of 18 countries, the mean serum 25(OH)D concentrations of 2606 osteoporotic postmenopausal women were 26.8 ng/mL (67 nmol/L) with an average serum 25(OH)D of 29.3 ng/mL (73.2 nmol/L) in Europe. Overall, 64 % of patients had serum concentrations < 30 ng/mL (75 nmol/L) and women receiving pharmacological treatment for osteoporosis had mean concentrations similar to those recorded in women who were not receiving such treatment [31]. In the Multiple Outcomes of Raloxifene Evaluation study, a large prospective intervention trial in postmenopausal women with osteoporosis, the mean serum 25(OH)D concentrations of the whole study population (n = 7564) were 70.8 nmol/L (65.9 nmol/L in Central Europe). Low serum 25(OH)D (<50 nmol/L) were observed in 39 % of the women living in Central Europe [32].

We also show that the prevalence of vitamin D inadequacy remains major, even amongst subjects receiving vitamin D supplements, with or without calcium. One could wonder if the supplementations are really appropriate to the individual needs in postmenopausal osteoporotic women although we have no data on the doses and length of supplementation used and the compliance of the subjects.

In our cohort, we observe a significant correlation between age and vitamin D [25(OH)D] concentrations. These results seem to be in accordance with previous studies, showing a highest prevalence of vitamin D deficiency in the oldest population varying from 5–25% in independent elderly to 60–80% in institutionalized elderly [32,33]. It has been shown that there is an age-dependent decrease in the epidermal concentrations of provitamin D3 (7-dehydrocholesterol) that explains the growing incapacity of human skin to produce vitamin D3 [10]. In elderly, kidneys are less effective to hydroxylate vitamin D to form 1,25-dihydroxyvitamin D (1,25(OH)2D3) [34]. Furthermore, there is a defect in intestinal absorption of cholecalciferol in the elderly, after oral ingestion [35]. In the same way, a late age-related decrease in calcium absorption is seen in postmenopausal women, in addition to the decline that occurs at menopause. This decrease could be due to a decline in either the active calcium transport or diffusion component of the calcium absorption system [36]. Moreover, in elderly, a depletion of body vitamin D stores results from a lack of sun exposure, due to progressive disability with age and subsequent the tendency to stay indoors, low dietary intake and reduced ability to adapt to a low calcium diet. This contributes to a higher prevalence of vitamin D inadequacy [34,37].

The combined deficiency in calcium and vitamin D, widespread in the ageing population, stimulates the secretion of parathyroid hormone which tends to normalize serum calcium concentrations, contributes to accelerated bone loss and plays a central role in pathogenesis of bone disorders and osteoporosis in the elderly [38]. Concomitantly, a decrease in bone strength and loss of muscle mass occurs.

Consistent with other reports, we find an inverse correlation between 25(OH)D and PTH, and a PTH rise with age [32,39,40]. In addition, 25(OH)D concentrations were positively correlated with hip BMD, similar to other studies of Caucasian postmenopausal women. The negative correlation of PTH concentrations with femoral neck BMD is also in accordance with other studies. [33,39,41-43]

In our study, we also show a difference in vitamin D [25(OH)D] concentrations between seasons, the lowest concentrations being seen during the spring months and the highest during the summer months. However, this difference is not statistically significant. These findings are in accordance with previous studies, especially with a similar study done in another European country [44]. In our study, it should be pointed that vitamin D inadequacy is high (over 89 % with the 80 nmol/L cut-off) even during summer. These results suggest that vitamin D supplement may be requested in osteoporotic postmenopausal women, independently of the season.

Many studies have demonstrated the effectiveness of calcium and vitamin D supplementation in fracture prevention particularly in patients with appropriate adherence to treatment. However, effectiveness of vitamin D alone in fracture prevention is still debated [45-52]. Calcium and vitamin D have been shown to reverse secondary hyperparathyroidism with resulting beneficial effects on bone mineral density (BMD)[37]. Additionally, it has been shown that calcium and vitamin D supplementation significantly improve body sway, muscle function and lower extremity strength, as well as reduce the risk of falls [53-55].

There is general agreement that adequate vitamin D and calcium intake is the cornerstone of osteoporosis prevention. In patients with documented osteoporosis, calcium and vitamin D supplementation should be a first line component of the osteoporosis care, along with antiresorptive or anabolic treatment [53].

## Conclusion

This study points out a high prevalence of vitamin D inadequacy in Belgian postmenopausal osteoporotic women even among subjects receiving vitamin D supplements. More studies are needed to address the amount of vitamin D intake necessary to maintain serum 25(OH)D to an adequate concentration which prevents secondary hyperparathyroidism and minimizes the possibility of further bone loss.

## Competing interests

The author(s) declare that they have no competing interests.

## Authors' contributions

AN and OB performed the statistical analysis, analyzed and interpreted the data. They drafted the manuscript. JYR revised it critically for important intellectual content. OB and JYR conceived of the study and participated in the design and coordination. All authors read and approved the final manuscript. JC performed the assays and critically analysed the screen samples.

## Pre-publication history

The pre-publication history for this paper can be accessed here:


